# Breast reconstruction after neoadjuvant radio chemotherapy: review and personal technique IDEAL concept REV-EJMR-D-15-00268

**DOI:** 10.1186/s40001-016-0219-8

**Published:** 2016-06-10

**Authors:** Carolin Nestle-Krämling, Edwin Bölke, Wilfried Budach, Christoph Andree

**Affiliations:** Department of Senology, Sana Kliniken Düsseldorf-Gerresheim, Graeulinger Straße 120, 40625 Düsseldorf, Germany; Department of Radiation Oncology, Heinrich Heine University, Moorenstrasse 5, 40225 Düsseldorf, Germany; Department of Plastic and Reconstructive Surgery, Sana Kliniken Düsseldorf-Gerresheim, Graeulinger Straße 120, 40625 Düsseldorf, Germany

**Keywords:** Neoadjuvant radio chemotherapy, Immediate breast reconstruction, Breast implant, Autologous breast reconstruction, DIEP flap, TRAM flap, IDEAL breast reconstruction

## Abstract

Neoadjuvant radio chemotherapy and immediate reconstruction for breast cancer are still under debate. But there are recent abstracts and articles which show that neoadjuvant radio chemotherapy is feasible and could improve the clinical outcome of breast cancer patients. The aim of this review is to present the authors’ techniques and approaches with regard to neoadjuvant radiation of breast cancer patients. It seems that the concept of immediate implant delayed autologous breast reconstruction could be a safe procedure that is at least equivalent to primary autologous reconstruction.

## Background

Neoadjuvant radio chemotherapy in breast cancer is still under debate. The concern of surgeons is that combined radio chemotherapy will affect the cosmetic results of their procedure and will cause severe side effects like impaired wound healing or fat necrosis. However, recent studies with long-term results, especially from the University of Dusseldorf, have shown that neoadjuvant radio chemotherapy is feasible and can improve the clinical outcome of breast cancer patients. This is in accordance with treatment modalities in other tumour entities like neoadjuvant radio chemotherapy for rectal or oesophagus cancer. Here, radio chemotherapy could demonstrate that the clinical outcome was not affected. In this review, we examine the actual status of neoadjuvant radio chemotherapy for breast cancer and investigate what kind of surgical procedures is available and the authors personal technique of the Ideal concept (immediate implant delayed autologous) for immediate breast reconstruction is presented.

## Review

The review was achieved by performing a pubmed and medline research using the search term neoadjuvant radio chemotherapy, breast cancer, cosmetic results and acute/late side effects, immediate breast reconstruction, breast implant, autologous breast reconstruction, DIEP flap, TRAM flap, and IDEAL breast reconstruction.

Neoadjuvant radio chemotherapy is a very interesting approach for breast cancer patients. It helps to reduce the tumour burden. Furthermore, there is an option to evaluate if the applied chemotherapy leads to a tumour reduction. Complete pathological response would be the best clinical result. This approach is in accordance with other tumour entities. In rectal cancer for T3 N+, neoadjuvant radio chemotherapy is the gold standard. With this procedure, the local relapse rate could be significantly reduced. With this approach and the mesorectal incision, the recurrence rate could be reduced below 10 %.

During 1991–1998, a total of 315 LABC (locally advanced breast cancer), patients (cT1-cT4/cN0-N1) were treated with neoadjuvant radio chemotherapy at the University of Dusseldorf. Preoperative radiotherapy (RT) consisted of external beam radiation therapy (EBRT) of 50 Gy (5 × 2 Gy/week) to the breast and the supra-/infraclavicular lymph nodes combined with an electron boost in 214 cases afterwards or—in case of breast conservation—a 10 Gy interstitial boost with (192) Ir after loading before EBRT. Chemotherapy was administered prior to RT in 192 patients and concomitantly in 113; 10 patients received no chemotherapy.

In 64 patients after breast conserving surgery and in 32 patients after mastectomy, a long-term follow-up of the cosmetic results was possible. Most patients rated their cosmetic results as excellent or good (80 % breast conserving surgery and 56 % mastectomy). After a follow-up of 14–21 years, we did not detect any grade III–IV fibrosis (24).

Skin-sparing mastectomy with immediate breast reconstruction (SSM-IBR) is increasingly used in invasive breast cancer. However, adjuvant chemotherapy (CT) and radiotherapy (RT) can increase the rate of local complications. Cécile Zinzindohoué et al. [25] published in 2016 a French prospective study in Annals of Surgical Oncology, which assessed the morbidity of SSM-IBR with latissimus and implant after neoadjuvant chemotherapy and radiotherapy. Among 94 patients included in this study, 83 were analysed (mean age 45.2 ± 9.5 years, T1 23.6 %, T2 55.6 %, T3 18.1 %). All but one patient received anthracyclines and taxanes and all patients received RT (49.3 ± 5.2 Gy) before SSM–IBR. Prostheses were used for IBR in 32 patients (mean volume 256 ± 73 mm^3^). Five patients had necrosis (≤2 cm, 2.2–10 cm^2^ and >10 cm^2^, in three, one and one cases, respectively) and they all recovered without revision surgery. Among 50 patients who underwent upfront mastectomy, 36 % achieved pCR. Like our results in this trial, neoadjuvant radio chemotherapy was safe, with an acceptable local morbidity rate.

The technique of nipple sparing mastectomy with immediate reconstruction is an oncologically safe procedure [13, 18, 22, 23] and associated with the best aesthetic results [6, 18] in case of therapeutic as well as of prophylactic mastectomies and if the postoperative course is uneventful. Autologous reconstruction by DIEP- or TRAM flap is associated with superior long time results [18, 24], but there are several surgery-associated complications which can severely impair long time results and oncologic outcome.

If a therapeutic mastectomy is planned as the primary cancer surgery simultaneously with a sentinel node biopsy, the tumour stage could be upgraded and the oncologic therapy regimen may switch to chemotherapy and post-mastectomy radiation even in cases that initially presented as in situ cancer by core needle biopsy. After complex oncologic and reconstructive surgery, a delay in adjuvant systemic therapy or radiation therapy cannot be excluded at least in single patients. But also in larger series and matched pair analysis, a significant delay of oncologic therapies was shown in patients with complications [2–4, 8, 11, 15, 21]. For immediate autologous reconstruction, the need for postmastectomy radiation after DIEP-or TRAM-flap surgery has the potential to deteriorate the aesthetic result, resulting in a fat fibrosis of the transferred tissue.

A further basic oncologic problem are positive resection margins in nipple sparing mastectomy specimen, especially in case of multicentric or extensive locally disease which are the most frequent indications for therapeutic mastectomy. In case of insufficient localization of the non-tumour-free margins, this can result in the need for post-mastectomy or post-reconstruction radiation, secondary modified radical mastectomy or acceptance of a significantly higher recurrence risk. Even if re-resection can be planned in case of clear assignment, it still will impair shape, scarring and overall appearance of the reconstructed breast. In case of R1 resection of the retro areolar region, the secondary excision of the nipple areolar complex (NAC) can be only compensated in autologous immediate reconstruction, if the flap was buried with the skin island and de-epithelialized in a second step after final histology.

Finally, the NSM technique is a highly demanding procedure in respect of skin and NAC perfusion, so that there will be a percentage between 2 and 22 % of skin or nipple necrosis. The rate of perfusion complications varies by surgical skills, mastectomy incision type [10, 15] and patients risk factors such as local factors like ptosis or breast hyperplasia with a need for mastopexy techniques. On the other hand, systemic factors like smoking, diabetes or obesity contribute to local complications. The combination of immediate autologous reconstruction and skin or NAC necrosis will end up in inferior aesthetic long-term results.

Currently, there are several published techniques to overcome problems associated with NSM/SSM and immediate reconstruction. The combination of implant surgery and the need of post-mastectomy radiation lead to higher rates of capsular contraction, reoperations, wound healing problems and implant loss [14, 16, 19, 20] and complication rates are slightly higher when radiation was before immediate reconstruction. In contrary in cases of complications of a complex reconstructive surgery before chemotherapy or radiation, there will be a higher probability of a delay of adjuvant oncologic therapies [5].

In case of indicated post-mastectomy radiation after immediate autologous reconstruction, the higher rate of volume loss, and fat necrosis due to tissue fibrosis [9, 12, 17] will lead to inferior results. With respect to recent publications [5], current guidelines [1], therefore, recommend autologous reconstruction to be performed after radiation therapy to avoid negative radiation effects on the healthy flap tissue.

On the other hand, implant reconstruction is recommended to be performed before radiotherapy to avoid wound healing problems of radiated tissue. For the majority of patients, it is impossible to decide as early as at the time of breast cancer diagnosis for either a definite heterologous or autologous reconstructive procedure: Furthermore, because in the standard oncologic approach for breast cancer surgery the result of the sentinel node biopsy is not clear, the planning of a primary reconstructive concept is difficult.

To avoid the coincidence of possible local oncologic or surgical ischaemic problems of NSM in the setting of autologous immediate reconstruction which could destroy the otherwise perfect long-term results of a highly specialized often microsurgical flap surgery, we decided to switch to an algorithm that is planned with an NSM and immediate breast reconstruction with implants only as the immediate implant delayed autologous concept (IDEAL concept). No immediate autologous reconstruction is done in the case of NSM, but the patient is fully informed of all options of implant based or autologous reconstructive procedures before ablative surgery. In case of uncertainty of the patients desire for autologous reconstruction or in case of confirmed patients desire for implant-based breast reconstruction, we offer a direct to implant reconstruction with mesh/matrix support (DTIMS) where the implant is placed partially subpectorally. If the patient opted for an autologous reconstruction, which is done mainly by uni- or bilateral DIEP flap procedure, she is counselled by the microsurgeon before the nipple sparing mastectomy and the implant is placed epipectorally at the end of the NSM procedure. Both surgical procedures of NSM with epi- or subpectoral implant positioning are performed by the specialized breast surgeon of the senology team.

**Fig. 1:**
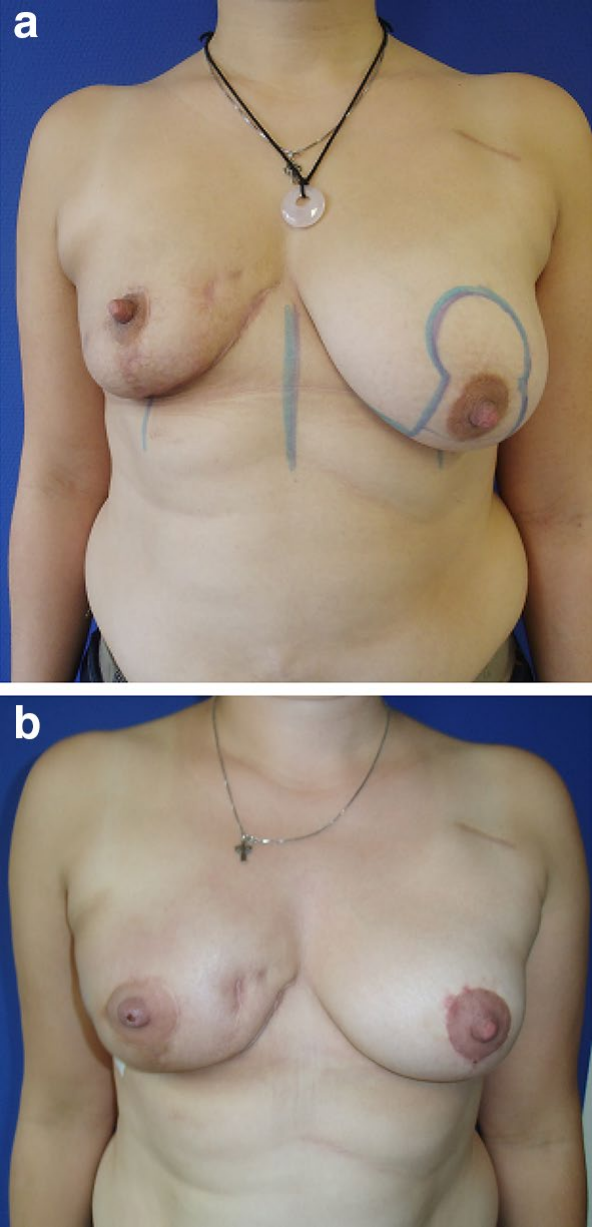
**a**–**b** Bilateral NSM with epipectoral implant positioning after oncoplastic breast conserving therapy and radiation therapy on the *right side* with consecutive breast deformity and significant asymmetry (**a**). A correction of the skin envelope was done by augmentation on the *right side* and inverted T skin reduction on the *left side* (**b**)

**Fig. 2:**
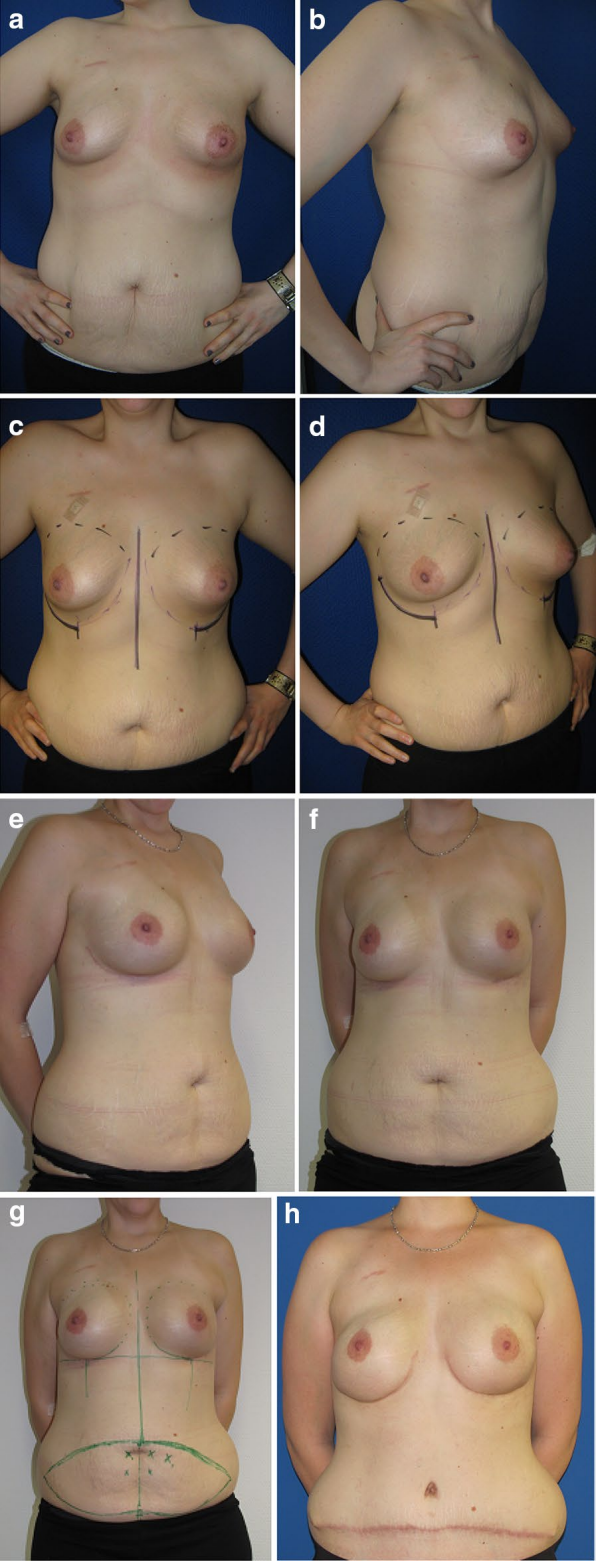
**a**–**h**: Patient with IBC on the *left side* and a BRCA mutation (**a**–**b**). After neoadjuvant chemotherapy, a bilateral NSM with correction of her tuberous breasts inframammary fold was planned (**c**–**d**) and an epipectoral implant reconstruction was performed (**e**–**f**). Implants were explanted and a DIEP flap was planned after 6 months (**g**–**h**)

**Fig. 3:**
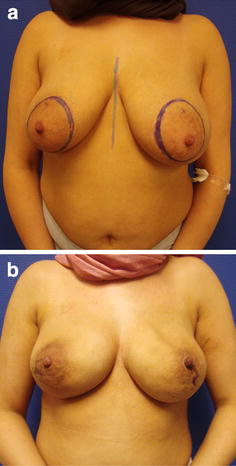
**a**–**b**: Result after bilateral NSM and epipectoral implant positioning (**b**) before planned DIEP flap. Note that the preoperatively planned periareolar NAC recentralization (**a**) was not done because of sufficient skin retraction at the end of the surgery

The personal technique of therapeutic or prophylactic nipple sparing mastectomy is combined with a simultaneous adjustment of the skin envelope whenever necessary in case of breast hyperplasia or ptosis, but in the majority of moderate skin excess the spontaneous breast skin retraction over the underlying implant is awaited. In case of therapeutic mastectomy intraoperatively, pretumoural re-resections are taken and in all cases of therapeutic or prophylactic mastectomy ventral re-resections are taken quadrant wise and from the retro areolar area. No frozen section was done but only the complete histologic report was seen as indication for resection of the NAC. The desired breast size can also result in reduction of large sized breasts as well as in a moderate augmentation (Fig. 1) and correction of other breast deformities as tubular breast. Mastectomy incisions are chosen whenever possible inferolaterally (Fig. 2a–h) and if necessary a superficial periareolar NAC re-centralization is added at the end of the surgery after the implant is in place. If the skin retraction is sufficient at the end of surgery, any additional lifting procedure is abandoned (Fig. 3). In cases of large breasts, an inverted T incision is planned for NSM with a craniocaudally NAC pedicle (Fig. 1).

If the patient has chosen an autologous reconstruction as her definite reconstructive procedure, the immediate implant reconstruction was done epipectorally, after reconstruction of the lateral breast contour by Rayen stitches. A drain was put in place and the optimal implant shape and size were chosen by mastectomy weight, diameter of breast base and filling of the skin envelope. A light circular bandage is placed in the operating room and changed to a bra without strong compression, which was worn for 6–8 weeks postoperatively. Drainages were kept until 20 ml per 24 h fluid production is seen. Prophylactic antibiotics were given as long as drainages were in place.

An uni- or bilateral DIEP flap or other autologous tissue transfer was done after complete wound healing, in the presence of oncological clear resection margins and a safe mastectomy skin and NAC perfusion 4–6 months later (Table 1). This microsurgical procedure was done by the plastic surgeon who is especially trained in microsurgery in a high-throughput department on a daily basis. Pedicle flap surgery was performed by the specialized breast surgeon of the senology department.

**Table 1:**
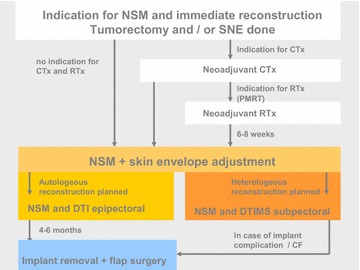
Algorithm of IDEAL technique: immediate implant delayed autologous reconstruction

*NSM* nipple sparing mastecomy; *SNE* sentinel node excision; *CTx* chemotherapy; *RTx* radiotherapy; *PMRT* post-mastectomy radiotherapy; *DTI* direct to implant; *DTIMS* direct to implant with mesh/matrix support; *CF* capsular fibrosis

If a patient decided for a definite implant reconstruction with mesh support and the long-term follow up is unsatisfying for the patient, the procedure and switch to a DIEP flap can be done in the same way. Implant and capsule/mesh were removed, the pectoralis muscle is sutured back to the thoracic wall and the DIEP flap was placed epipectorally in the former implant pocket.

If the resection margins are not free of tumour at any area after the nipple sparing mastectomy specimen was examined by the pathologist, a localized re-resection was planned around 4–6 weeks postoperatively in any case, where tumour free margins could be achieved with a high probability. In cases of non-tumour-free margins in the retroareolar region, the NAC is resected and according to the amount of skin removed, the implant is changed to a tissue expander (TE).

The same manoeuver was done in cases of skin or NAC ischaemia and failure of conservative treatment [7]. After waiting some weeks for demarcation of necrosis, the region was excised and the implant was replaced by TE.

Due to the fact that oncologic therapies could be significantly delayed by complications of complex reconstructive surgery [3, 4, 8, 21], we examine the nodal status by either sentinel lymph node excision or axillary dissection whenever indicated. The consequences for any indication for chemotherapy or post-mastectomy radiation are then confirmed or excluded and all oncologic adjuvant therapies are planned in a neoadjuvant setting before NSM and immediate implant reconstruction. The surgery is planned as early as 6–8 weeks after the end of the radiation therapy (Table 1).

## Conclusion

It seems that neoadjuvant radio chemotherapy is a safe and reliable method in breast cancer patients. One interesting approach is the concept of immediate implant, delayed autologous breast reconstruction in all cases of nipple sparing mastectomy to receive optimal oncologic therapy sequence as well as optimal aesthetic results.
