# Erratum to: Breast reconstruction after neoadjuvant radio chemotherapy: review and personal technique IDEAL concept

**DOI:** 10.1186/s40001-016-0223-z

**Published:** 2016-07-14

**Authors:** Carolin Nestle-Krämling, Edwin Bölke, Wilfried Budach, Christoph Andree

**Affiliations:** Department of Senology, Sana Kliniken Düsseldorf-Gerresheim, Graeulinger Straße 120, 40625 Düsseldorf, Germany; Department of Radiation Oncology, Heinrich Heine University, Moorenstrasse 5, 40225 Düsseldorf, Germany; Department of Plastic and Reconstructive Surgery, Sana Kliniken Düsseldorf-Gerresheim, Graeulinger Straße 120, 40625 Düsseldorf, Germany

## Erratum to: Eur J Med Res (2016) 21:24 DOI 10.1186/s40001-016-0219-8

After publication of the original article [1], it was noticed there was an error in the article title. The article title was incorrectly presented as: “Breast reconstruction after neoadjuvant radio chemotherapy: review and personal technique IDEAL concept REV-EJMR-D-15-00268”. The correct article title is included in this erratum and has been updated in the original article [1].

In addition to this error, it was noticed one of the references had been duplicated. Reference 23 [2] was duplicated and was also included as Reference 25. Reference 25 was removed from the original article [1] and confirmation of Reference 23 [2] has been included in this erratum.

There were also edits made to the original article [1] in the last sentence of the Abstract. Originally, this was presented as: “It seems that the concept of immediate implant delayed autologous breast reconstruction could be a safe procedure that is at least equivalent to primary autologous reconstruction,” however, the capitalisation of the IDEAL concept has been updated in the original article [1] accordingly. The updated sentence in the Abstract now reads: “It seems that the concept of Immediate implant DElayed AutoLogous breast reconstruction (IDEAL breast reconstruction) could be a safe procedure that is at least equivalent to primary autologous reconstruction.”

A similar edit was also made to the last sentence of the ‘Background’ section in the original article [1]. The sentence was originally presented as: “In this review, we examine the actual status of neoadjuvant radio chemotherapy for breast cancer and investigate what kind of surgical procedures is available and the authors personal technique of the Ideal concept (immediate implant delayed autologous) for immediate breast reconstruction is presented.” However, capitalisation of the IDEAL concept was updated, so the sentence now reads: “In this review, we examine the actual status of neoadjuvant radio chemotherapy for breast cancer and investigate what kind of surgical procedures is available and the authors personal technique of the IDEAL concept (immediate implant delayed autologous) for immediate breast reconstruction is presented.”
